# Brainstem activation of GABA_B_ receptors in the nucleus tractus solitarius increases gastric motility

**DOI:** 10.3389/fnins.2022.961042

**Published:** 2022-08-02

**Authors:** Lorenza Bellusci, Elizabeth Kim, Selena Garcia DuBar, Richard A. Gillis, Stefano Vicini, Niaz Sahibzada

**Affiliations:** Department of Pharmacology and Physiology, Georgetown University Medical Center, Washington, DC, United States

**Keywords:** vagus, patch-clamp, optogenetic, DMV, brainstem, NTS

## Abstract

**Background and aim:**

Local GABAergic signaling in the dorsal vagal complex (DVC) is essential to control gastric function. While the inhibitory GABA_A_ receptor action on motility in the DVC is well-documented, the role of the GABA_B_ receptor on gastric function is less well-established. Microinjection of baclofen, a selective GABA_B_ receptor agonist, in the dorsal motor nucleus of the vagus (DMV) increases gastric tone and motility, while the effect on motility in the nucleus tractus solitarius (NTS) needs to be investigated. Previous *in vitro* studies showed that GABA_B_ receptors exert a local inhibitory effect in unidentified NTS neurons. Since the NTS and DMV nuclei have differential control of gastric motility, we compared GABA_B_ receptor activation in the NTS to that reported in the DMV. We microinjected baclofen unilaterally in the NTS while monitoring intragastric pressure and compared its action to optogenetic activation of somatostatin (SST) neurons in transgenic *sst*-Cre::channelrhodopsin-2 (ChR2) mice. We also performed patch-clamp recordings from SST and DMV neurons in brainstem slices from these mice.

**Methods:**

*In vivo* drug injections and optogenetic stimulation were performed in fasted urethane/α-chloralose anesthetized male mice. Gastric tone and motility were monitored by an intragastric balloon inserted in the antrum and inflated with warm water to provide a baseline intragastric pressure (IGP). Coronal brainstem slices were obtained from the *sst*-Cre::ChR2 mice for interrogation with optogenetics and pharmacology using electrophysiology.

**Results:**

The unilateral microinjection of baclofen into the NTS caused a robust increase in gastric tone and motility that was not affected by ipsilateral vagotomy. Optogenetic activation of SST neurons that followed baclofen effectively suppresses the gastric motility *in vivo*. In brain slices, baclofen suppressed spontaneous and light-activated inhibitory postsynaptic currents in SST and gastrointestinal-projection DMV neurons and produced outward currents.

**Conclusion:**

Our results show that GABA_B_ receptors in the NTS strongly increase gastric tone and motility. Optogenetic stimulation *in vivo* and *in vitro* suggests that these receptors activated by baclofen suppress the glutamatergic sensory vagal afferents in the NTS and also inhibit the interneurons and the inhibitory neurons that project to the DMV, which, in turn, increase motility *via* a cholinergic excitatory pathway to the stomach.

## Introduction

The gamma-aminobutyric acid) GABA(signaling in the dorsal vagal complex (DVC) of the brainstem is critical to the regulation of the receptive relaxation reflex of the stomach (see review, Gillis et al., 2021). This nuclear complex (Chang et al., 2003; Travagli et al., 2003, 2006; Fan et al., 2020; Feng et al., 2020; Li et al., 2021) includes the nucleus tractus solitarius (NTS) and dorsal motor nucleus of the vagus (DMV), which are differentially modified *via* GABA receptors to control the increase or decrease of gastric motility and tone (Gillis et al., 2021). Notably, inhibition of GABA_A_ receptors in the NTS profoundly decreases gastric motility (Ferreira et al., 2002; Herman et al., 2009), whereas, in the DMV, this increases its activity (Ferreira et al., 2002; Herman et al., 2009). In the NTS, two major inhibitory projection pathways (GABAergic and noradrenergic) to the DMV selectively influence the gastric tone and motility and are closely monitored and controlled by GABA interneurons (Ferreira et al., 2002, 2005; Davis et al., 2004; Rogers et al., 2006; Pearson et al., 2007, 2011; Herman et al., 2008). Interrupting the activity of GABAergic interneurons permits the inhibitory projection pathways to the DMV to decrease the gastric response contractions (see recent review, Gillis et al., 2021). It is the opposite of the DMV, where impeding GABAergic afferents disinhibit the pacemaker-like cholinergic neurons projecting to the stomach, thus increasing motility (Gillis et al., 2021).

In addition to GABA_A_ receptor signaling, GABA_B_ receptors also influence neurotransmission (Bowery et al., 1987; Enna and Bowery, 2004; Chalifoux and Carter, 2011). In the DVC, GABA_B_ receptors appeared to be important for the regulation of gastric motility (McDermott et al., 2001; Cruz et al., 2019). Microinjection of baclofen, a GABA_B_ receptor agonist, in the DMV of rats and systemic administration in ferrets increases gastric motility through the excitatory cholinergic vagal pathway (Cruz et al., 2019). Interestingly, the application of baclofen on gastric DMV neurons elicited outward currents (Browning and Travagli, 2001), which would potentially diminish vagal outflow to the stomach. To reconcile these observations, we need to consider that *in vivo* application of baclofen in the DMV presynaptically blocks inhibitory projection neurons from the NTS and somatostatin (SST) interneurons in the DMV (Lewin et al., 2016; Cruz et al., 2019). Hence, this would allow the “decoupling” of the neurons to inhibit gastric DMV neurons and suppress motility. Moreover, baclofen's effect on outward currents in the DMV neurons is concentration-dependent (Browning and Travagli, 2001). In addition, it has been reported that presynaptic release on NTS neurons is more sensitive to baclofen compared to postsynaptic response (Brooks et al., 1992).

To further understand the contribution of GABA_B_ receptors in the DVC on gastric motility, we investigated how the NTS differentially regulated stomach contractions *via* the DMV (Gillis et al., 2021). Specifically, we focused on GABA interneurons, namely SST neurons in the nucleus that gate an extensive network that regulates viscerosensory inputs (Thek et al., 2019), thus exhibiting a capacity to play a critical role in integrating and modulating the vagovagal reflex. In the DVC, these neurons differently affect gastric motility by increasing it in the NTS and decreasing it in the DMV (Lewin et al., 2016; Bellusci et al., 2022).

We report here that *in vivo* microinjection of baclofen in the NTS of SST transgenic mice similarly enhanced gastric motility as did its injection in the DMV of the rat (Cruz et al., 2019), which was decreased following injection of L-glutamate in these nuclei (Gillis et al., 2021). L-glutamate in the NTS decreases gastric contractions compared to the application in the DMV, where it increases activity. Baclofen increased stomach contractions which were not blocked by ipsilateral vagotomy, showing that the response originated in the NTS. These contractions were attenuated by CGP5845 and gabazine, antagonists of GABA_B_ and GABA_A_, respectively.

In brainstem slices from *sst*-Cre::ChR2 mice, application of baclofen suppressed spontaneous inhibitory postsynaptic currents (sIPSCs) in both the SST neurons of the DVC and gastric projections neurons of the DMV. Furthermore, synaptic responses evoked by optogenetic activation of the neurons were attenuated. Moreover, optogenetic activation of SST neurons in the DMV is followed by the suppression of gastric motility (Bellusci et al., 2022). Taken together, these *in vivo* and *in vitro* results demonstrate that activation of GABA_B_ signaling in NTS *via* SST neurons mirrored baclofen motility increase in the DMV, as suggested in Cruz et al. (2019). Our study will determine the role of GABA_B_ signaling in the NTS considering all the possible targets that include SST neurons, inhibitory projection neurons from the NTS to the DMV, and the release of glutamate from vagal afferent neurons.

## Materials and methods

### Animals

All animals were housed in a climate-controlled animal facility (22 ± 2°C) and maintained on a 12-h light/dark cycle with *ad libitum* access to food and water. Animal housing rooms were maintained at MP14 barrier (pathogen and opportunistic free) in the animal facility before experiments. Transgenic SST mice, referred to as *sst*-Cre::ChR2 in this study, were all The Jackson Laboratory “reporter” strains Sst-Cre (RRID:IMSR_JAX:013044); Rosa-ChR2-EYFP (RRID:IMSR_JAX:012569) either sex 1–2 months old. All procedures in this study followed the National Institutes of Health (U.S.A.) guidelines, U.K. regulations for the ethical use of animals in research (Drummond, 2009), and the approval of the Georgetown University Animal Care and Use Committee.

### *In vivo* recording

Before all experiments, food was withheld for 4 h, but water *ad libitum*. Animals were anesthetized with an intraperitoneal (I.P.) injection containing a mixture of urethane (1,000 mg/kg) and α-chloralose (60 mg/kg) dissolved in 0.9% saline. Body temperature was maintained at 37 ± 1°C with an infrared heat lamp.

### Surgical preparation

After the depth of anesthesia was confirmed by lack of pedal and corneal reflexes, mice were intubated *via* the trachea following a tracheotomy to maintain an open airway and institute artificial respiration when necessary. Both cervical vagi were carefully isolated from each carotid artery on either side and looped with a 5-0 silk thread for later avulsion during the experiment.

Subsequent to the cervical vagi loop, a laparotomy was performed to expose the stomach. An intragastric balloon (made from the tip of a latex condom ~0.3 cm long) was inserted into the stomach *via* the fundus and positioned in the antrum's distal region, secured in place by a purse-string suture. The balloon was inflated (with warm water ~100–150 μl) to produce a baseline pressure of 3–6 mmHg, which was measured by a pressure transducer (sensitivity: 5 μV/V/mmHg) that was connected to it as described by Richardson et al. (2013) for monitoring blood pressure in rats. The abdominal cavity was closed at the end of the procedure using a gut suture.

### Microinjection procedure

Each anesthetized mouse was placed prone in an animal stereotaxic frame (Kopf Instruments, Tujunga, CA). Before stereotaxic surgery, each animal was pretreated with dexamethasone (0.8 mg, S.C.) to minimize swelling of the brain. A skull-based partial craniotomy was performed to expose the dorsal medulla. The cerebellum was retracted to gain access to the entire rostrocaudal extent of the NTS and DMV that underlie, and the underlying dura was cut and reflected to expose the NTS.

The DVC is a small-scale area (anteroposterior 1.2 mm, mediolateral 0.3 mm, and dorsoventral 0.6 mm dimensions) and is relatively fibrous in the medulla as defined in our review (Gillis et al., 2021). To assess this fibrous area, we successfully made a microfiber-stainless optic cannula (50–70 μm, Doric Lenses Inc) to penetrate this to activate to interrogate the tone and motility of the stomach. We also have assembled this optic cannula with four-barreled glass pipettes (tip Ø = 30–70 μm; Frederick Haer, New Brunswick, ME), which can microinject pharmacological solutions into the NTS or the DMV.

The optic cannula and four-barreled glass pipettes (30° angle from the perpendicular) were inserted into the NTS or DMV using the caudal tip of the area postrema, the *calamus scriptorius* (CS), as a reference point for determining the coordinates for micropipette placement. Stereotaxic coordinates for the optic fiber cannula into the NTS were as follows: *AP* = +0.3–0.5 mm rostral to C.S.; *ML* = 0.1–0.3 mm lateral to the midline; and *DV* = 0.0–0.1 mm ventral to the dorsal surface of the medulla. Coordinates for the DMV were as follows: +0.3–0.5 mm rostral to C.S.; 0.1–0.3 mm lateral to the midline; and 0.2–0.3 mm ventral to the dorsal surface of the medulla.

The precise location of the NTS and the DMV for optogenetic induced responses was also assessed by microinjecting L-glutamate (500 pmol/30 nl) into the sites. The L-glutamate induced opposite gastric motility responses from nuclei; in NTS, it decreases, whereas DMV increases these responses (Ferreira et al., 2002).

Drugs were loaded and ejected from each barrel by negative or positive pressure, respectively, through a 5-ml syringe connected *via* a PE-50 polyethylene tubing. Drug injections into the NTS were administered manually within 5 to 10 s in a 30 nl volume, as monitored by a calibration tape affixed to the pipette.

### Histological verification of pipette tracks

The animal was euthanized at the end of each experiment. The brain was removed and fixed in a solution of 4% paraformaldehyde and 20% sucrose for at least 24 h. It was then cut on a cryostat into 50 μm-thick coronal serial sections and stained with 0.5% neutral red. Microinjection sites for the cannula tracks' locations were photographed and were identified using the mouse brain's atlas (Paxinos and Franklin, 2007).

### Optogenetic procedure

Optogenetic in *sst-*cre::ChR2 mice was performed using an envelope (90 or 60 s) photostimulation by trains of light pulses (frequency 15 Hz, pulse width 10 or 40 ms, duty cycle of 15 or 60%) with a laser (λ 473 nm; 1.20 μW; Shanghai Laser & Optics Century Co., China) that was controlled by a Master-8 stimulator (AMPI, Israel) connected the PowerLab data acquisition system (ADInstruments, Colorado Springs, Co). These optogenetic stimulation parameters are compatible with those used in respiration studies (Abbott et al., 2013).

### Drugs

Drugs used for the studies were urethane, a-chloralose, dexamethasone sodium phosphate, L-glutamate, baclofen HCL, bicuculline methobromide (BMR), and atropine methyl bromide. All drugs were purchased from Sigma-Aldrich (St. Louis, MO) except dexamethasone and BMR, which were purchased from American Regent Laboratories (Shirley, NY) and Hello Bio, respectively. All drug solutions were constituted in 0.9% saline (pH 7.2–7.4).

### Experimental design and statistical analysis

Analysis of the intragastric pressure recordings was completed “off-line” using the Chart (ADInstruments) and Prism (GraphPad Software, Inc.) software packages. The experimental recordings were initially filtered using a root mean square (RMS; 2 s moving window) algorithm to account for respiration and various other signal artifacts. All peaks were selected within the 90 or 60 s of optogenetic stimulation and compared to the immediately prior peaks, selected for the same amount of time (baseline). An algorithm for calculating average peak-to-peak values was used to determine a change in the amplitude of gastric phasic contractions. This algorithm was used because gastric phasic contractions have a low frequency of occurrence. Due to the natural variance in the amplitude of gastric contractions, the average peak-to-peak values proved to be a uniform method to compare all datasets. However, following analysis of peak-to-peak parameters of baseline values and those measured during optogenetic stimulation found that differences in an animal's intrinsic activity could introduce considerable variance in the results. Optogenetic stimulation response was induced, and each animal was normalized to its baseline value to solve this problem, which was displayed as a percent change in the gastric contractions. The noise level on all completed peak-to-peak analyses was set to zero as an RMS algorithm had already filtered the data. Baseline and optogenetic stimulation peak values (amplitude; AMP) were taken as a percent difference using the following formula: (((optogenetic stimulation-baseline)/(baseline)) x100). The same formula has been used to calculate the gastric tone (area under the curve of the contractions; AUC).

### Quantification and statistical analysis

Statistical analyses were performed using Prism 9 (GraphPad, La Jolla, CA). All data indicated in the text and figures were checked for normal distribution, and the results were expressed as means ± S.E.M. unless otherwise stated. The statistical analysis comparisons between two groups were performed by paired Student's *t*-test or unpaired *t*-test with Welch's correction. Comparisons among three or more groups were performed with one-way variance analysis (ANOVA) followed by Holm–Šídák's *post-hoc* test, Brown–Forsythe test, and Welch test, followed by Dunnett's T3 multiple comparisons test or using mixed-effects model followed by Tukey's multiple comparisons test. All percentages from *in vivo* data were analyzed for the significance of normality using the D'Agostino and Pearson omnibus and or Shapiro–Wilk normality test. A one-sample *t*-test was performed on the data with the hypothetical value set at zero. For ipsilateral vagotomy data, a two-sample paired *t*-test was performed. Animal numbers and cell numbers are indicated in the text.

### Electrophysiology

Neurons were identified visually by infrared-differential interference contrast (IR-DIC) or episcopic fluorescence optics and a CMOS camera (Thorlabs). All recordings from SST and gastrointestinal-projection DMV neurons [monosynaptically labeled with DiI applied to the antrum of the stomach (Lewin et al., 2016; Bellusci et al., 2022)] were mainly in the NTS and DMV. A 60x-water immersion objective was used for identifying and approaching neurons. Recordings were made with patch electrodes (5–6 M*Ω*; Warner Instruments, Hamden, CT) with internal pipette solution (pH 7.2; 285 mOsm) that was composed of the following (in mM): 145 K-KCl 5 EGTA, 5 MgCl2, 10 HEPES, 5 ATP-Na, and 0.2 GTP-Na. Whole-cell voltage-clamp recordings were performed using a MultiClamp 700B amplifier (Molecular Devices, Sunnyvale CA). A 5 mV hyperpolarization pulse monitored input and access resistances; series resistance was typically < 10M*Ω* and was not compensated. Resting membrane potentials were corrected for liquid junction potential for the KCl intracellular solution (−3mV). Signals were low-pass filtered at 2 kHz and acquired with a Digidata 1440A (Molecular Devices, Sunnyvale CA).

### *In vitro* optogenetic stimulation

Light stimulation of coronal brainstem slices was accomplished utilizing filter cubes of the microscope (ChR2, l = 450–490 nM) *via* white light from an X-Cite 120 LED (Excelitas Technologies, Waltham, MA). Slices were excited with a maximal light intensity adjusted to prevent loss of voltage clamp (Vhold = −70 mV). The diameter of the area exposed to optogenetic stimulation under the 60 x objective was < 100 μm and encompassed the whole DVC area.

### Drugs

Stock solutions of the following drugs were prepared in water and purchased from the following companies: D(–)-2-Amino-5-phosphonopentanoic acid (AP-5, Cat#3693, Tocris), bicuculline methobromide (B.M.R., Cat#HB0894 Hello Bio), 2,3-Dioxo-6-nitro-1,2,3,4-tetrahydrobenzo[f]quinoxaline-7-sulfonamide disodium salt (NBQX, Cat#ab120046, Abcam), R&D Systems (Minneapolis, MN); tetrodotoxin (T.T.X., Cat#HB1035, Hello Bio); 4-Aminopyridine **(**Cat # A-0152, Sigma-Aldrich), kynurenic acid Na salt, (Cat. # 3694 Tocris Bioscience), CPG55845 hydrochloride (Cat # 1248 Tocris Bioscience stock concentration 5 mM), and gabazine (SR 95531 hydrobromide, Cat # 1262, Tocris Bioscience, stock concentration 10 mM). Drug-containing stock solutions were diluted to desired concentrations in either ACSF. All drugs were applied *via* the “Y-tube” application adapted to brain slices (Murase et al., 1989; Hevers and Lüddens, 2002).

### *In vitro* data analysis

Electrophysiological data were analyzed off-line using pClamp 11 (Molecular Devices, Inc.). A semi-automated threshold pClamp 11 software was employed with 5–10 times the baseline noise depending on the voltage clamp (Vhold = −60 mV) to analyze inhibitory postsynaptic currents. The control and treatment data were acquired from two consecutive 500 ms segments. During optogenetic activation in *sst*-Cre::ChR2 mice while recording from SST neuron or gastric antrum projection DMV neurons the voltage-clamp mode was set at −60 mV in the presence of NBQX (5 μM) and AP-5 (10 μm). IPSCs were seen as downward deflections (inward currents). We were able to clearly visualize a one-to-one correlation between the onset of the optogenetic stimulation and its effect on the postsynaptic current.

## Results

We investigated the GABA_B_ signaling in the NTS and its effects on gastric motility and SST neurons in *sst*-Cre::ChR2 mice using *in vivo* and *in vitro* studies.

### Microinjection of baclofen in the NTS increases gastric motility

**Figure 1:**
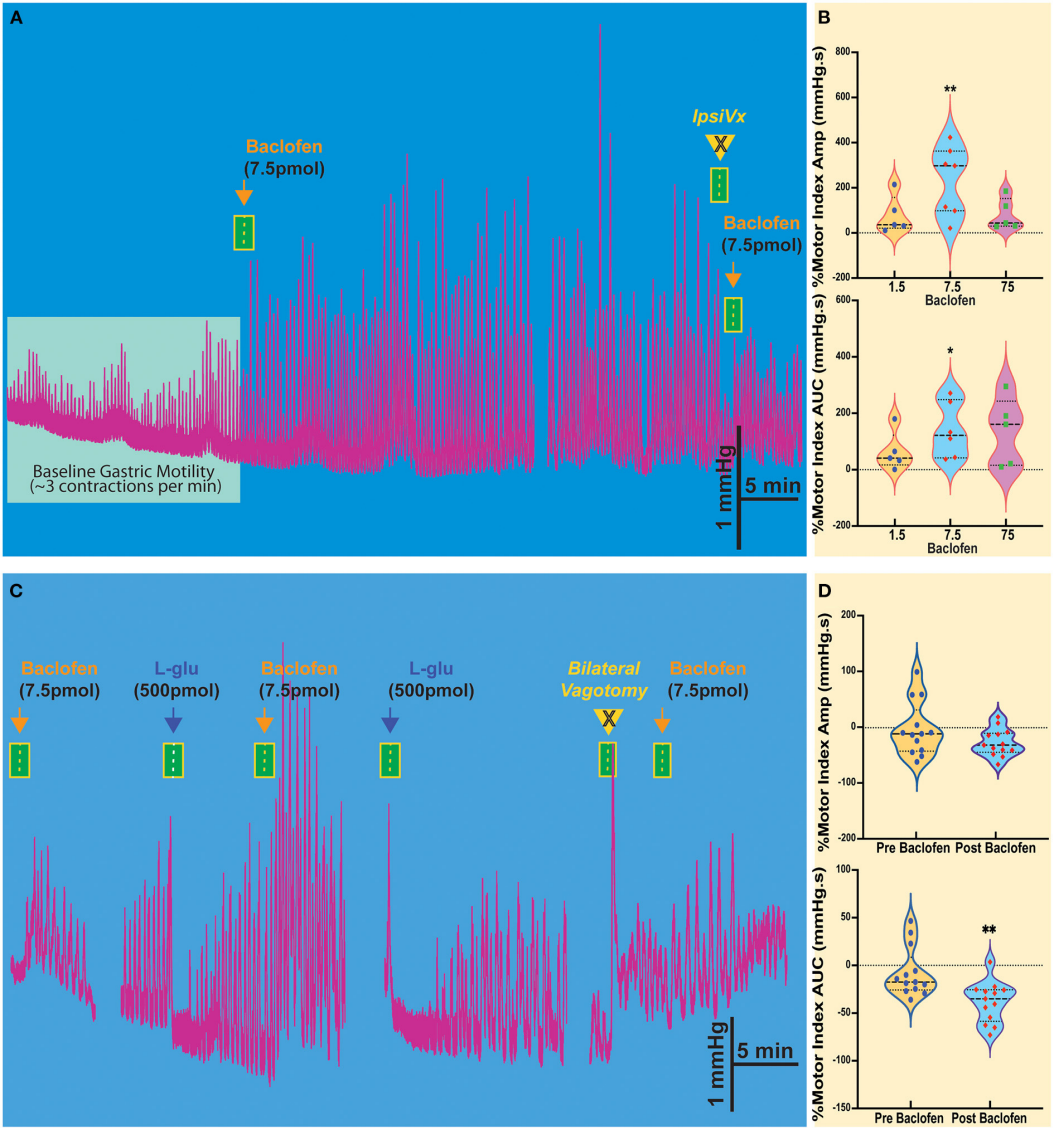
Baclofen microinjection into the NTS of the Sst-ChR2 mouse causes a robust and prolonged increase in gastric contractions. **(A)** The enhanced gastric motility induced by baclofen injection is attenuated ipsilateral vagotomy (IpsiVx) but not blocked. **(B)** Dose responses of percentage changes in motor index of amplitude (AMP) and area under the curve (AUC). *p = 0.0699 and **p = 0.018. **(C)** Microinjection of L-glutamate in the NTS produces a well-known temporary decrease of gastric motility. The baclofen-mediated increase of gastric motility is blocked by bilateral vagotomy. **(D)** % of reduction of motor index amplitude (AMP) and motor index area under the curve (AUC) induced by L-glutamate microinjection before and after baclofen microinjection. **p = 0.0396.

Injection of baclofen (7.5 pmol/30 nl), a GABA_B_ agonist, significantly increases the intragastric pressure of the stomach (IGP; Figure 1A), has been reported after baclofen injection in the DMV of rats (Cruz et al., 2019). After baclofen injection (7.5 pmol/30 nl), the amplitude of IGP increased by 231.7 ± 57.58 mmHg.s %motor index; *p* = 0.0069) from the baseline (Figure 1B, Table 1). The area under the curve (AUC) of the motor index was significantly higher than the baseline (139.0 ± 40.08 mmHg.s %motor index; *p* = 0.018; Figure 1B, Table 1). Because of the proximity with the underlying DMV, we did an ipsilateral vagotomy to ensure that the baclofen effect on motility originated in the NTS (Ferreira et al., 2002, 2005; Cruz et al., 2007; Herman et al., 2009, 2010; Richardson et al., 2013). Since the NTS projects bilaterally to the DMV (e.g., see Norgren, 1978; Kalia and Sullivan, 1982) and, in turn, send outs premotor neurons to the stomach, vagotomy allows us to differentiate between NTS and DMV responses; while the ipsilateral vagotomy prevents only the response of the ipsilateral DMV, bilateral vagotomy is needed to block also the effect of the NTS (Ferreira et al., 2002, 2005; Cruz et al., 2007; Herman et al., 2009, 2010; Richardson et al., 2013). Ipsilateral vagotomy attenuated the enhancement of gastric motility induced by baclofen injection in the NTS (AMP 184 ± 60.2 mmHg.s; *p* = 0.1219; AUC 88.2 ± 53.2 mmHg.s, *p* = 0.0626; *n* = 5 mice), but it did not significantly block the response (Figure 1A, Table 1). However, in the anesthetized mice, ipsilateral vagotomy depresses cardiorespiratory systems to the extent that gastric motility responses cannot be accurately evaluated. Nevertheless, we used ipsilateral vagotomy at the end of each experiment to ensure that its treatment effect on gastric responses is the NTS or the DMV origins. Hence, gastric responses elicited from the NTS are blocked by ipsilateral vagotomy as in the DMV (Cruz et al., 2019).

**Table 1:** Pharmacological and optogenetic control of gastric motility (MMI%) and tone (AUC%) in the NTS.

**Drug 1**	**Drug 2**		***N***	**Mean ±SEM**	**t**	**df**	***p***	**Test**
***Baclofen 1.5***		*Motility*	5	78.4 ± 37.1	2.111	4	0.1023	Two-tailed, one sample *t*-test
		*Tone*	5	64 ± 30.7	2.082	4	0.1058	Two-tailed, one sample *t*-test
***Baclofen 7.5***		*Motility*	7	231.7 ± 57.6	4.03	6	0.0069	Two-tailed, one sample *t*-test
		*Tone*	6	139.0 ± 40.1	3.57	5	0.018	Two-tailed, one sample *t*-test
	Ipsi Vx	*Motility*	5	184 ± 81.0	2	4	0.1219	Two-tailed, paired *t*-test
		*Tone*	5	88.2 ± 53.2	2.02	4	0.0626	Two-tailed, paired *t*-test
	L-Glutamate	*Motility*	13	−35.6 ± 5.9	3.275	12	0.0396	Two-tailed, paired *t*-test
		*Tone*	13	−0.29 ± 13.1	2.653	12	0.021	Two-tailed, paired *t*-test
	GABAzine	*Motility*	7	−35.7 ± 4.55	4.210	6	0.0056	Two-tailed, paired *t*-test
		*Tone*	7	−45.24 ± 3.0	6.1423	6	0.0009	Two-tailed, paired *t*-test
***Baclofen 75***		*Motility*	5	81.6 ± 30.76	2.653	4	0.0568	Two-tailed, one sample *t*-test
		*Tone*	5	135.6 ± 53.8	2.519	2	0.0654	two-tailed, one sample *t*-test
***SST Chr2***		*Motility*	5	73.0 ± 33.3	2.194	4	0.0093	Two-tailed, one sample *t*-test
		*Tone*	5	48.5 ± 9.6	5.028	4	0.0073	Twoo-tailed, one sample *t*-test
	GABAzine	*Motility*	5	−97.0 ± 46.9	2.754	8	0.0249	Two tailed, paired *t*-test
		*Tone*	5	−72.1 ± 18.6	4.186	4	0.0139	Two tailed, paired *t*-test
	Baclofen	*Motility*	3	20.8 ± 32.3	3.652	2	0.0675	Two tailed, paired *t*-test
		*Tone*	3	12.4 ± 13.1	0.2229	2	0.8443	Two tailed, paired *t*-test
	CGP	*Motility*	3	−1.6 ± 9.7	3.538	2	0.0714	Two tailed, paired *t*-test
		*Tone*	3	−18.2 ± 18.5	2.659	3	0.1171	Two tailed, paired *t*-test
***CGP55845***		*Motility*	4	54.7 ± 8.2	6.698	4	0.0068	Two-tailed, one sample *t*-test
		*Tone*	4	39.5 ± 18.7	2.113	3	0.125	Two-tailed, one sample *t*-test

A summary of in vivo results obtained with pharmacological regulation of optogenetic control in SST Chr2 mice.

The activation of the NTS or DMV induces opposite responses in gastric motility. Stimulation of the NTS by L-glutamate decreases gastric tone and motility, whereas, in the DMV, it increases this response (cf., Ferreira et al., 2002; Cruz et al., 2007; Herman et al., 2009; Richardson et al., 2013). In the NTS, we have shown that these distinctive responses are due to GABAergic and noradrenergic DMV-projection neurons that affect the gastric parasympathetic neurons (see review, Gillis et al., 2021). Since baclofen injection in the NTS imitates the increased gastric response elicited from the DMV, we investigated whether GABA_B_ receptors attenuate the inhibitory DMV-projection neurons (Cruz et al., 2019). As illustrated in Figure 1C, baclofen injected in the NTS robustly increased gastric motility, being temporarily inhibited by L-glutamate. These inhibitory effects on gastric motility can be replicated. Figure 1D and Table 1 report changes in AMP and AUC induced by L-glutamate microinjection after baclofen microinjection (AMP-0.29 ± 13.1 mmHg.s *p* = 0.021; AUC −35.6 ± 5.9 mmHg.s, *p* = 0.0396; *n* = 13 mice). The bilateral vagotomy prevented these gastric responses of the NTS (not shown). In all animals, confirmation of the micropipette sites of the NTS was histologically verified.

These results show that activation of GABA_B_ receptors in the NTS resembles the strong effect of baclofen seen with gastric motility in the DMV (Cruz et al., 2019). These suggest the presence of GABA_B_ receptors prominently on NTS neurons that affect presynaptic disinhibition of neurons (Brooks et al., 1992; Herman et al., 2009, 2012; Cruz et al., 2019; Gillis et al., 2021) by uncoupling of inhibitory neurotransmission from NTS to DMV neurons that modulate gastric motility (Gillis et al., 2021; Bellusci et al., 2022). This uncoupling status is briefly halted by L-glutamate injection in the NTS by exciting the inhibitory neurons that project to DMV.

### Microinjection of GABA_A_ antagonist on baclofen-induced gastric motility in the NTS

**Figure 2:**
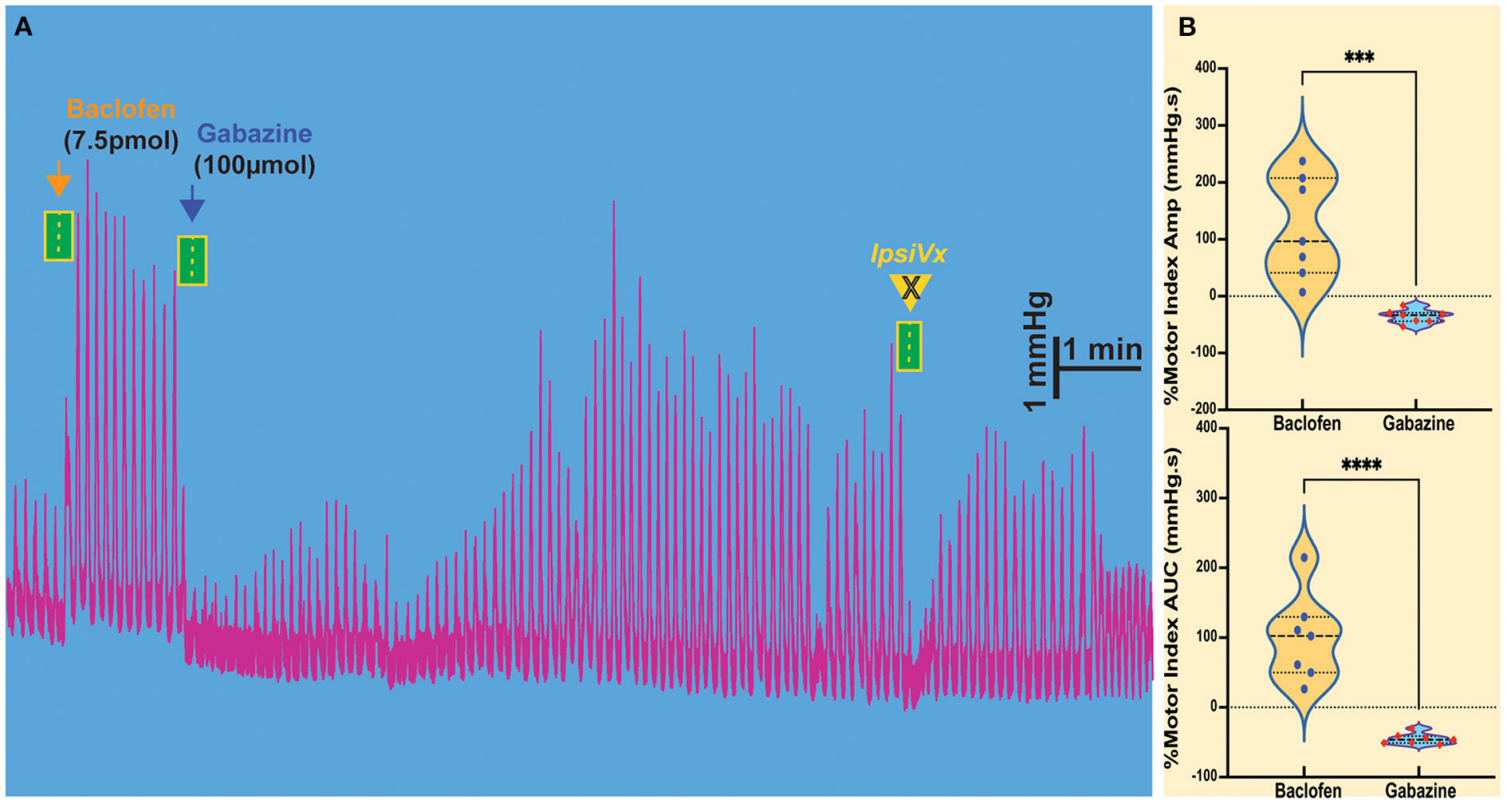
Modulation of gastric motility by GABA_B_ and GABA_A_ receptors in the NTS. **(A)** The excitatory gastric contractions induced by microinjection of baclofen are suppressed following microinjection of gabazine, a GABA_A_ antagonist, but are not blocked by ipsilateral vagotomy (IpsiVx). **(B)** Percentage of motor index amplitude (AMP) and area under the curve (AUC) after baclofen injections (7.5pmol/ 30nl) into the NTS and followed by gabazine injection. ***p = 0.0056 and ****p = 0.0009.

The injection of baclofen in the NTS invariably increases gastric motility. We wanted to study the action of subsequent exposure to gabazine, an antagonist of the GABA_A_ receptor, to verify whether local GABAergic signaling in the NTS was susceptible to the activity of GABA_B_ receptors. Our previous studies demonstrated that inhibition of GABA_A_ signaling in the NTS exerted the same effect of L-glutamate injection on DMV-projection neurons, showing that these neurons are subservient to local GABAergic interneurons (Ferreira et al., 2002; Herman et al., 2009; Gillis et al., 2021). As previously observed with L-glutamate, gabazine injected into the NTS caused a decrease in motility (AMP −35.71 ± 4.55 mmHg.s *p* = 0.0056; AUC −45.24 ± 3.00 mmHg.s *p* = 0.0009; *n* = 7 mice; Figures 2A,B, Table 1) that was initially enhanced by baclofen. Similarly, to the effect of L-glutamate (Ferreira et al., 2002; Herman et al., 2009; Gillis et al., 2021), these gastric responses could not be blocked by ipsilateral vagotomy (as mentioned above).

### Interaction of GABA_A_ and GABA_B_ receptors on SST-GABA neurons

**Figure 3:**
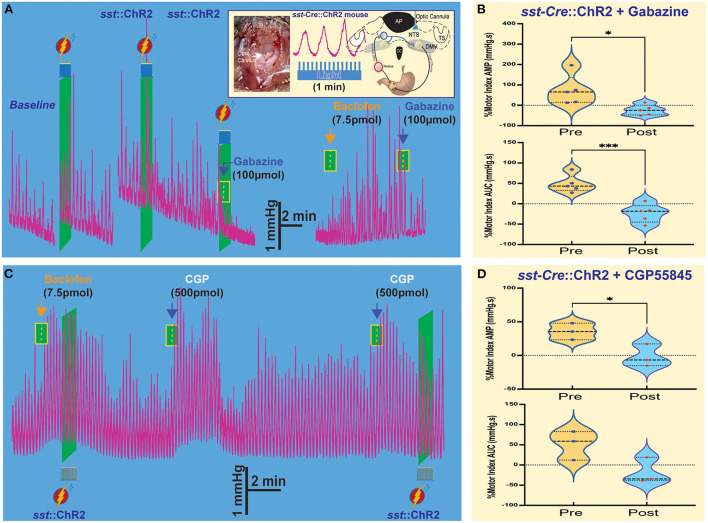
Optogenetic stimulation of SST neurons combined with microinjections in the NTS affects gastric tone and motility. **(A)** Gastric contractions are increased by optogenetic stimulation of SST neurons in the NTS of *sst*-Cre::ChR2 mice. The effect is abolished after the gabazine administration. Insert: stereotaxic location and hindbrain approach of optogenetic stimulation. **(B)** Percentage of motor index AMP and AUC due to optogenetic stimulation of SST neurons in NTS of *sst*-Cre::ChR2 mice before and after gabazine. *p = 0.0249 and ***p = 0.0139. **(C)** Baclofen increase of gastric tone and motility is not affected by optogenetic stimulation, but subsequent administration of CGP5845, a GABA_B_ receptor antagonist, also increases gastric tone and motility. **(D)** Percentage of motor index AMP and AUC due to optogenetic stimulation of SST neurons in NTS of *sst*-Cre::ChR2 mice before and after GCP5845 microinjection. *p = 0.0068. AP, area postrema; CC, central canal; DMV, the dorsal motor nucleus of the vagus; NTS, nucleus tractus solitarius; TS, solitary tract.

We used *Sst-cre* transgenic mice (Taniguchi et al., 2011) bred with a floxed-stop channelrhodopsin-2 (ChR2) mouse to obtain *sst-*Cre::ChR2 mice (Lewin et al., 2016; Bellusci et al., 2022). These mice allow us to evaluate the relationship between GABA_B_ receptors and SST neurons in the NTS and its effects on gastric motility. To optogenetically stimulate Chr2-expressing SST neurons, we used trains of 60 s light pulses (pulse train = 15; frequency = 15 Hz; pulse duration = 40 ms; power ~9–12 mW) to reliably produce the response (Figure 3A Insert) without any danger of tissue lesion (Senova et al., 2017) [Note: gastric motility is ~3–6 contractions per min]. Because the DVC is a small-scale area (~anteroposterior 1.2 mm, mediolateral 0.3 mm, and dorsoventral 0.6 mm dimensions), we assembled four-barreled glass pipettes with a stainless-fiber optic cannula by which we could both microinject pharmacological solutions into the NTS and optogenetically stimulate the same exact area.

As illustrated in the example in Figure 3A, light-induced activation of SST neurons induced a robust increase in tone and motility (Figure 3B, Table 1). Subsequently, administration of gabazine suppressed gastric responses by light-induced activation of SST interneurons (Figure 3A, Table 1). Gabazine also abolished gastric responses induced by baclofen administration. In contrast, light-induced activation did not affect the baclofen response (Figure 3C, Table 1). On the contrary, pre-administration of CPG55845, an antagonist of GABA_B_ receptors *per se*, also increases gastric contractions (% motor index of AMP 54.7+16.34 mmHg.s; *p* = 0.0068 and AUC 39.47 + 37.35 mmHg.s; *p* = 0.1250; Figure 3C, Table 1). However, optogenetic activation increase in % motility index for AMP was abolished by CGP5845 (AMP −1.60 ± 9.68 mmHg.s; *p* = 0.0714; AUC −18.2 ± 18.5, *p* = 0.117 mmHg.s; *n* = 3 mice; Figure 3D, Table 1). These findings suggest that GABA_B_ receptors may play a significant role in the regulation of SST neurons that control gastric motility (Gillis et al., 2021). Microinjection of kynurenic acid, a glutamate receptor antagonist that does not exert any effect, *per se*, on gastric motility if injected in the NTS (Herman et al., 2009), increased gastric responses after CPG55845 application (data not shown). This implies the essential role of glutamate (from the nodose afferents) and GABA signaling (on GABA_B_ receptors) in the NTS area in the modulation of gastric activity. Both kynurenic acid and CGP5845 block the DMV-projection neurons and SST neurons' inhibitory “brake” on these neurons. This transmission brake allows spontaneously firing pacemaker-like gastrointestinal-projection neurons in the DMV to increase contractions (see, e.g., Richardson et al., 2013).

### GABA_B_ signaling on SST and DMV neurons

To understand how GABA_B_ signaling increased gastric activities, we investigate the role of SST neurons and DMV-gastric projection neurons in the NTS. The SST neurons are essential to vagovagal reflex control and the accommodation reflex of the stomach by gastrointestinal neurons in the DMV (Gillis et al., 2021; Bellusci et al., 2022).

**Figure 4:**
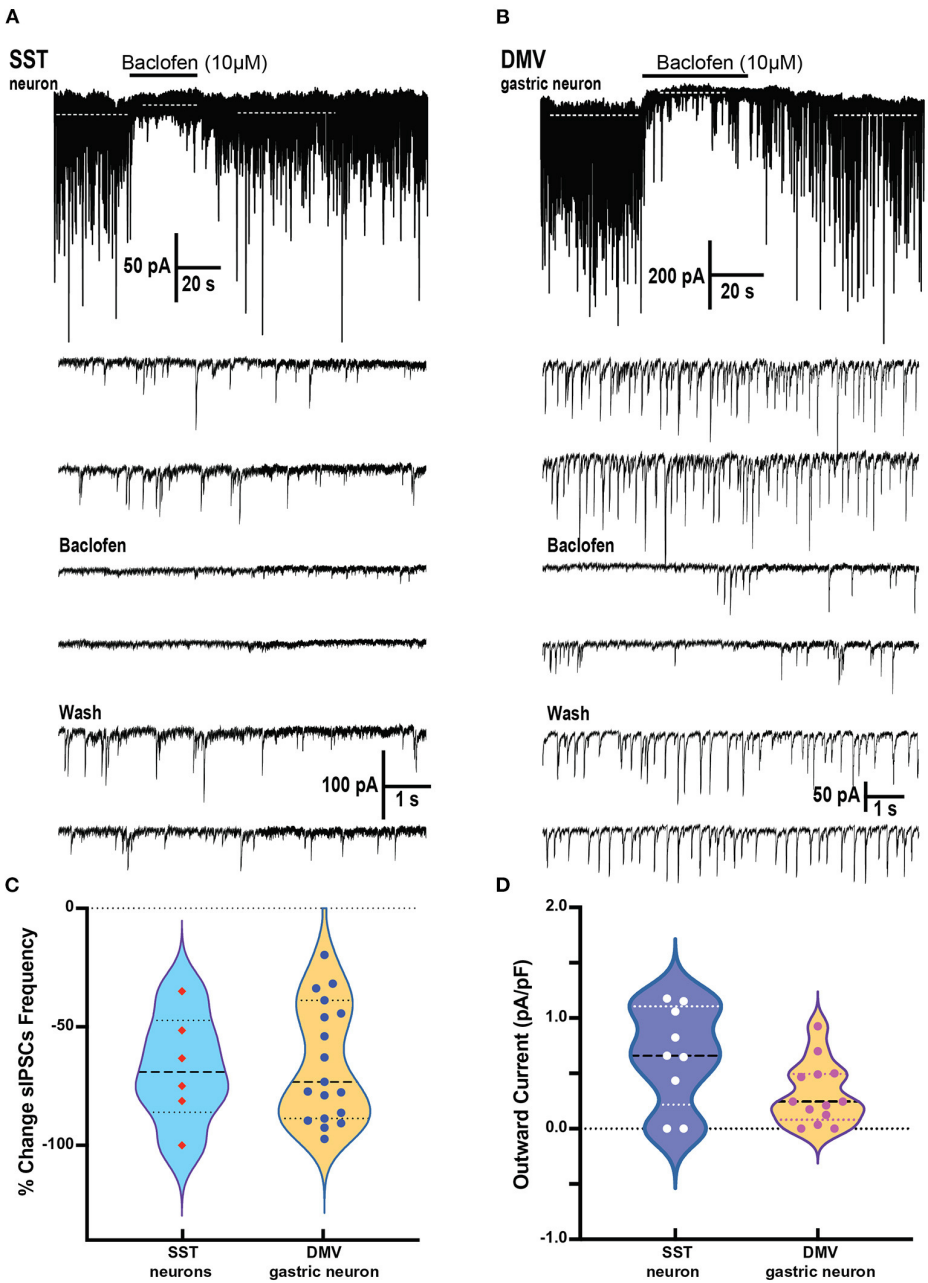
Baclofen inhibits spontaneous inhibitory postsynaptic currents and produces outward current in SST and gastrointestinal-projection DMV neurons of the DVC. **(A,B)** Representative recording of action local (y-tube) baclofen perfusion on whole-cell currents in a SST **(A)** and a gastrointestinal-projection DMV neuron **(B)**. Top panel shows with a compressed time scale a strong reduction of synaptic activity and the occurrence outward currents (dashed white lines). Bottom panels: the sIPSCs frequency and amplitude are inhibited in both neurons. **(C,D)** Summary % change in sIPSCs frequency **(C)** and the outward currents normalized to cell capacitance **(D)** with the application of baclofen on SST and gastrointestinal-projection DMV neurons.

Brainstem slices were obtained from mice with an SST-Cre::Chr2 (10 μM) on SST neurons which produced a significant decrease in the frequency of sIPSCs from a baseline of 1.64 ± 0.65 to 0.56 ± 0.31 Hz, respectively (*p* = 0.02 paired *t*-test; 6 cells from 4 mice; Figures 4A,C). This change in the frequency of sIPSCs was −67.7 ± 9.4% of control (Figure 4C). The frequency decline did not allow for assessment changes in sIPSCs amplitude, although the sIPSC amplitude in two out of six cells with remaining events was not decreased. In all six SST cells tested, BMR (25 μM) abolished sIPSCs.

To identify the gastrointestinal-projection neurons in the DMV, we recorded from neurons monosynaptically labeled with DiI applied to the antrum of the stomach. Similar to the SST neurons, sIPSCs occurrence was profoundly inhibited by baclofen (10 μM; Figure 4B). The baseline of sIPSCs frequency significantly decreased in the presence of baclofen from 3.31 ± 0.57 to 1.18 ± 0.2 Hz, respectively (*p* = 0.0009; paired *t*-test 20 cells, nine mice). The sIPSC amplitude in these cells was also reduced significantly (baseline 56.5 ± 9.5 to baclofen 35.2 ± 5.4 pA, respectively; *p* = 0.0138 paired *t*-test). The percent reductions in sIPSCs frequency and peak were 60.6 ± 6.3% and 28.4 ± 6.2%, respectively (Figure 4C). sIPSCs were abolished by BMR in all DMV neurons as well. A comparison between the reduction in sIPSCs frequency between DMV and SST neurons did not reveal significant differences (Kolmogorov–Smirnov test).

In addition to depressing sIPSC, baclofen produces outward currents in SST and DMV neurons (Figures 4A,B) that are mediated by G-coupled inwardly rectifying potassium (GIRK) channels mediated by GABA_B_ receptors (Browning and Travagli, 2001). In SST neurons, the outward current normalized to cell capacitance produced by baclofen application was 0.66 ± 0.15 pA/pF *p* < 0.02 (Figure 4D) in the cells tested (nine cells from five mice). Baclofen-induced outward currents in 13 gastrointestinal-projection DMV neurons from five mice averaged 0.32 ± 0.08 pA/pF (Figure 4D). A comparison of outward current density between DMV and SST neurons did not reveal significant differences (Mann–Whitney test).

### Interactions of SST and DMV neurons on GABA_B_ signaling

**Figure 5:**
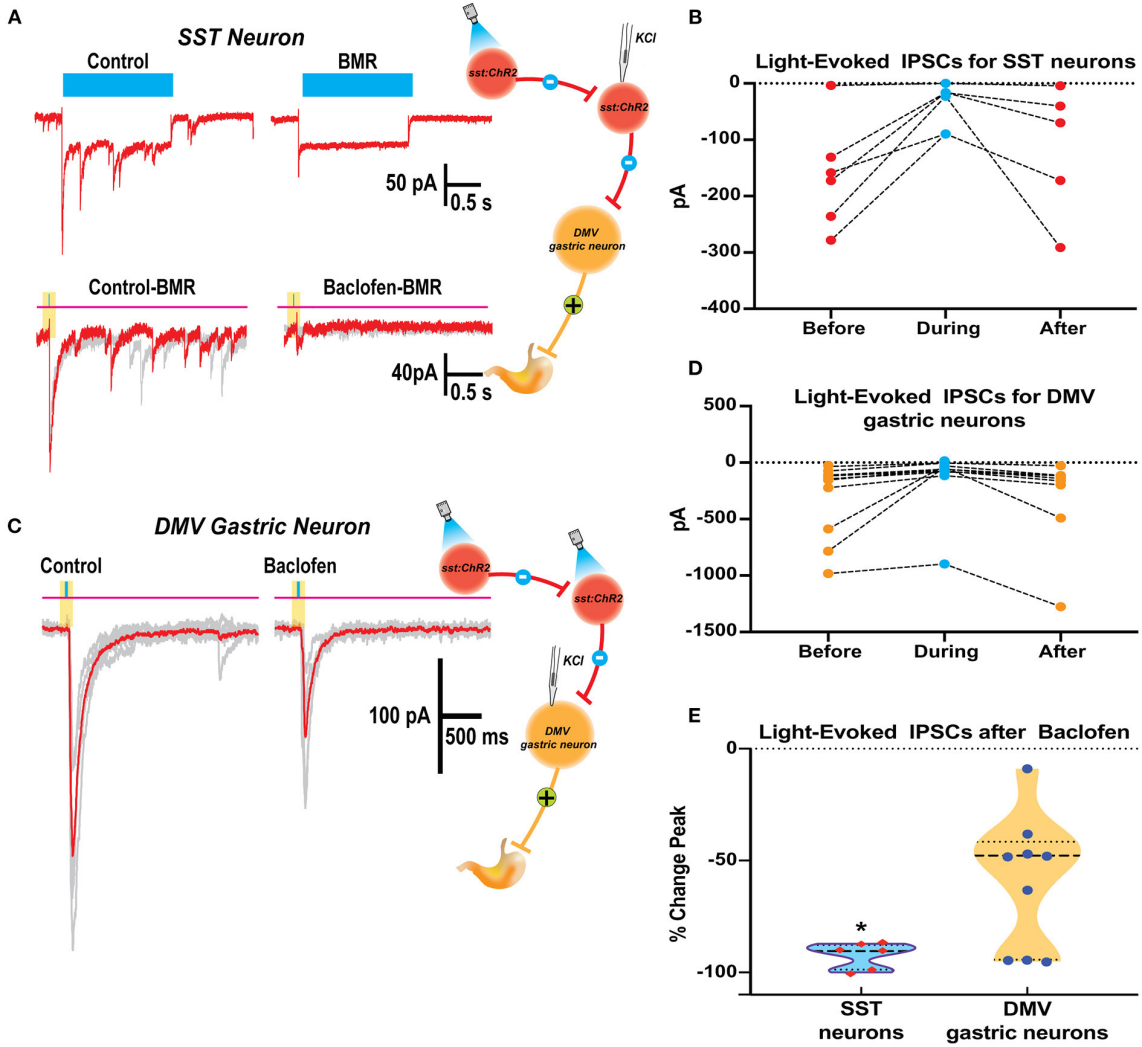
Baclofen suppression of light evoked IPSCs in SST and DMV gastric neurons in the DVC of SST-cre:ChR2 mice. **(A)** Current elicited in a Chr2 expressing SST neuron by a 2 s blue light reveals ChR2 direct inward current with overlapping light evoked IPSCs (*top left*) that are blocked by perfusion with BMR (*top right*). BMR subtracted responses (20ms light application) before (*bottom left*) and during baclofen (*bottom right*) illustrates inhibition of synaptic activity. **(B)** Summary results with light activation of IPSCs before, during, and after baclofen perfusion in the SST neurons studied. **(C)** Light-activation of IPSCs in a DMV gastric neuron before and during baclofen exposure. **(D)** Summary results with light activation of IPSCs before, during, and after baclofen perfusion in the DMV neurons studied. **(E)** Violin plot of the % change in peak for IPSCs of SST and DMV neurons studied. *p < 0.05.

We examined the effects of baclofen on the response to optogenetic stimulation of SST neurons in brain slices from *Sst*-*Cre*::*Ch2R* mice in SST and gastric DMV neurons (Figures 5A,B). Since activating channelrhodopsin-2 channels induce per se light-induced current in SST neurons, we used BMR to subtract these currents and study the effect of baclofen. Subtraction of the trace in BMR from control (Figure 5A top panels) revealed overlapping light-evoked IPSC from reciprocal connections between SST neurons. Two subtracted traces superimposed in red and gray in control and baclofen are shown for SST neurons (Figure 5A, bottom panel). The light-evoked IPSCs in SST were significantly attenuated by baclofen from control (−92.4 ± 2.1% change, paired *t*-test *p* = 0.012; six cells three mice; Figure 5B).

In gastrointestinal DMV neurons, baclofen depressed light-evoked IPSCs evoked by the optogenetic stimulation of SST neurons (−57.9 ± 9.12%, *p* = 0.0160 paired *t*-test 10 cells five mice; Figure 5D). However, the reduction of the light-evoked IPSCs was significantly larger in SST than in DMV neurons (*p* = 0.0042 *t*-test with Welch's correction). The percent change in peak for IPSCs of SST and DMV neurons studied is compared in Figure 5E.

The strong inhibitory action by GABA_B_ signaling of SST neurons predominately located in the NTS may lead to the suppression of inhibitory DMV-projection neurons in the NTS and underlie an increase in the neural activity of gastrointestinal neurons in the DMV that are responsible for contractions of the stomach as observed *in vivo*.

## Discussion

The GABAergic signaling in the DVC is pivotal to regulate the gastric vagovagal reflex activity (Gillis et al., 2021). Previously, we found with *in vivo* pharmacological studies that the activation of the GABA_B_ receptors of the DMV increased gastric motility as did the suppression of direct NTS inhibition to the DMV (Cruz et al., 2019). Our study extends those findings on controlling gastric contractions by the GABA_B_ receptors in the NTS. We also took advantage of optogenetics in mice that allows light-induced activation of SST neurons, abundantly present in the DVC (Lewin et al., 2016; Thek et al., 2019; Bellusci et al., 2022) to focus on how GABA_B_ receptors contribute to gastric vagovagal reflex via these neurons.

Activation of GABA_B_ receptors in the NTS increases gastric motility. These findings are based on the results of administration of the agonist of the GABA_B_, baclofen, *in vivo* and *in vitro*. In the *in vivo* model, baclofen: (1) prominently enhances contraction of the stomach, an effect not blocked by ipsilateral vagotomy; (2) restores IGP motility after L-glutamate or gabazine injections, which transiently inhibit the response; and (3) increased gastric motility is not enhanced by optogenetic stimulation of SST neurons.

Brainstem slices in the *in vitro* preparation showed that baclofen: (1) robustly inhibits sIPSCs on SST neurons in the NTS and gastrointestinal neurons in the DMV; (2) produces larger outward currents in gastrointestinal neuron in the DMV than in the SST neurons; and (3) produces a stronger blockade of light-evoked IPSCs induce by optogenetic stimulation in SST-Chr2 mice in synaptically coupled SST neurons than in gastrointestinal neuron in the DMV.

### *In vivo* activation of GABA_B_ signaling in NTS increases gastric motility

Previously, we showed that direct stimulation of NTS or DMV induces distinct differential responses (e.g., review of Gillis et al., 2021). Briefly, activation of the NTS causes a decrease in motility, whereas, in the DMV, it increases this response.

**Figure 6:**
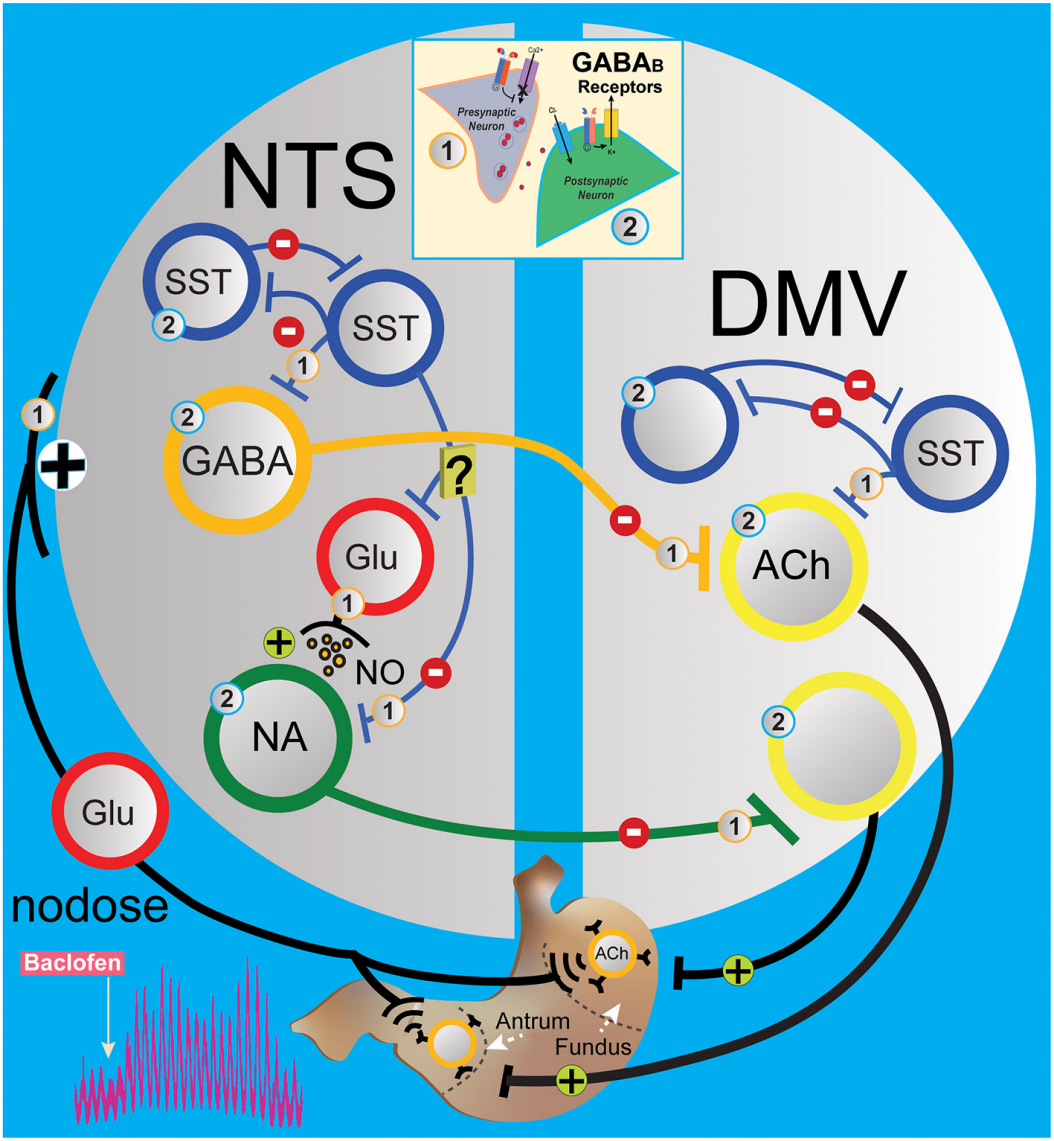
Simplified circuit illustrating how GABA_B_ signaling in the DVC may modify gastric motility and tone. The activation of presynaptic **(1)** and postsynaptic **(2)** GABA_B_ receptors (see insert) G protein coupled with voltage-gated Ca^2+^ channels and inward rectifying potassium (GIRK) channels, respectively, to regulate neurotransmission in the gastric vagovagal circuits of the DVC. In NTS, sensory vagal information from the periphery excites *via* the nodose ganglia local and projection neurons in the NTS. This information is integrated and conveyed back to the stomach *via* a vagal efferent reflex loop arising from the DMV. Baclofen in the NTS acts at presynaptic **(1)** and postsynaptic **(2)** GABA_B_ receptors to inhibit interneurons (blue and red) and projections inhibitory neurons (GABAergic and noradrenergic, orange and green) to the gastrointestinal-projection DMV neurons (yellow). Local GABA signaling by SST neurons in the DVC (blue) is at the centerpiece controlling one of the circuits that operate during the stomach's receptive relaxation reflex. SST neurons in NTS are secondary neurons that suppress projections of inhibitory neurons to the DMV, which allow contractions of the stomach *via* releasing pacemaker activity of gastrointestinal-projection neurons. Baclofen in the NTS inhibits SST and projection inhibitory neurons along with nodose glutamatergic afferents, which permits the “self-spontaneous activating” cholinergic neurons that project to the stomach to increase motility. [Note: In the DMV, baclofen is effectively hyperpolarized gastrointestinal-projection DMV neurons *via* GIRK channels, but presynaptic **(1)** GABA_B_ receptors have been reported to be more sensitive to baclofen compared to postsynaptic **(2)** GABA_B_ receptors]. ACh, acetylcholine; DMV, dorsal motor vagal nucleus; Glu, glutamate; NA, noradrenergic; NTS, nucleus tractus solitarius; SST, somatostatin neurons.

In the NTS, the decrease in gastric motility is essentially due to the activation of DMV-projection GABAergic and noradrenergic neurons in the nucleus that leads to the suppression of DMV excitatory neurons to the stomach (Ferreira et al., 2002; Herman et al., 2009, 2012; Gillis et al., 2021). Microinjection of baclofen in the DMV caused a robust dose-dependent increase in gastric tone and motility that was abolished by ipsilateral vagotomy and counteracted by pretreatment with atropine (Cruz et al., 2019). This result indicates that baclofen injected in the DMV acts on presynaptic GABA_B_ receptors from the inhibitory NTS neurons that bilaterally terminate on DMV neurons, thereby uncoupling excitatory gastric cholinergic neurons in the DMV from an inhibitory tone, causing an increase in gastric tone and motility. Our results with *in vivo* application of baclofen in the NTS rapidly increase gastric tone and motility (as seen in Figure 1). L-glutamate transient inhibition of the baclofen effects suggests a role for inhibitory DMV-projection neurons in the NTS to terminate gastric contraction. However, glutamatergic signaling is essential for the operation of the vagal circuitry as blockade in the NTS *per se* does not affect gastric mechanical activity (Herman et al., 2009). Instead, the overwhelming interneurons' GABAergic drive in the NTS controls the level of activity in the vagovagal circuit (Herman et al., 2009, 2012) by a glutamatergic input reflex from the nodose on the SST neurons and inhibitory DMV-projection neurons (GABA and noradrenergic) in the NTS (Figure 6) that terminate to the DMV (Glatzer et al., 2007; Gao et al., 2009; Thek et al., 2019). As illustrated in Figure 6, in the NTS, this interplay of local GABAergic drive on DMV-projection neurons (inhibitory) with nodose glutamatergic input (excitatory) leads to an enhancement or a reduction of the intragastric pressure of the stomach *via* gastric DMV neurons. The suppression of GABAergic signaling in these nuclei has the opposite effect on gastric motility.

Interestingly, the increased effect on gastric motility by baclofen is also seen with CGP5845, the antagonist of GABA_B_ receptors. We propose a model in Figure 6 suggesting that CPG55845 antagonizes a tonic activation of GABA_B_ receptor in the NTS. This activation enhances vagal glutamatergic afferent neurons to the inhibitory DMV-projection neurons but also to the SST interneurons (Glatzer et al., 2003; Thek et al., 2019) and NPY neurons (Chen et al., 2020) in NTS. We propose that the balance between these pathways is tilted in favor of SST neurons that inhibit GABAergic DMV-projection and/or excitatory NPY neurons that we have shown to activate DMV neurons (Bellusci et al., 2022). As baclofen has the same effect on gastric motility, we propose that the suppression of vagal glutamatergic afferent neurons to the inhibitory DMV-projection neurons is predominant.

Gabazine, a GABA_A_ antagonist, affects baclofen-induced gastric motility as seen in Figure 2 (inhibiting local GABAergic neurons control of DMV-projection neurons), as with L-glutamate administration (directly exciting DMV-projection neurons in the NTS). This result indicates that local GABA_A_ neurons are primarily involved in baclofen-induced gastric responses in the NTS, as seen in *in vitro* studies (see below). This is not surprising (Figure 6) since inhibitory DMV-projection neurons in the NTS are subservient to the activity of the predominant SST-GABA interneurons in the NTS (Herman et al., 2009, 2012; Lewin et al., 2016; Thek et al., 2019; Bellusci et al., 2022). The baclofen-evoked increase in stomach contractions comes essentially from suppressing the nodose afferents and SST interneurons (presynaptic receptors, as noted by number 1 in Figure 6), which cannot excite inhibitory DMV-projection neurons in the NTS. They permit the intrinsic spontaneous firing frequency of cholinergic gastrointestinal-projection DMV neurons (Pedarzani et al., 2000) to enhance contractions by uncoupling from extrinsic inhibitory NTS neurons. In the NTS, gabazine blocks baclofen effect on motility; thus, we propose that SST neuron efferents are transiently inactivated by the GABA_A_ antagonist, disconnecting GABAergic terminal drive to an inhibitory projection, tilting the excitation inhibition balance of vagovagal reflex, and, thus, impeding excitatory gastrointestinal-projection DMV neurons activity.

Moreover, the inhibition of many agonist substances, for instance, melanocortin or μ-opioid receptors, allows a decrease in gastric motility due to the uncoupling of NTS inhibitory DMV-projection neurons to suppress the excitatory gastrointestinal neurons of the DMV (Herman et al., 2010; Richardson et al., 2013; Lewin et al., 2016).

Our data highlight previous work on the importance of GABA_B_ signaling of local GABAergic neurons in the gastric vagal circuit (Travagli et al., 1991; Kawai and Senba, 1996; Smith et al., 1998; Glatzer et al., 2003; Bulmer et al., 2005; Bailey et al., 2008; Herman et al., 2009; Gillis et al., 2021). We provide further evidence that identifiable SST neurons in transgenic SST-cre mice (Lewin et al., 2016; Thek et al., 2019) in the DVC play a crucial role in regulating the DVC vagovagal gastric circuit (Lewin et al., 2016; Gillis et al., 2021; Bellusci et al., 2022).

Optogenetic activation of SST neurons in the NTS, after baclofen-induced activity, did not alter the baclofen-induced increase in gastric contractions that was followed by inhibition of gabazine, as seen in Figure 3C (also, see Bellusci et al., 2022). This finding further supports the key role for SST neurons in the baclofen response. It also support the role of the proposal that the activation of GABA_B_ receptors affects both SST neuron inhibition of NTS projection neurons and attenuates glutamatergic transmission from nodose afferents silencing the NTS to DMV information flow and allowing gastrointestinal-projection DMV neurons to resume driving stomach contraction. This hypothesis is corroborated by the action of the GABA_B_ antagonist on the effect of kynurenic acids, which *per se* does not increase motility. Indeed, the GABA_B_ antagonist acts similarly to the GABA_A_ antagonist in the NTS, increasing stomach contractions (Herman et al., 2009), but *via* distinct presynaptic (GABA_B_) and postsynaptic (GABA_A_) pathways. The increase in gastric contractions is due to uncoupling gastrointestinal-projection DMV neurons that are excitatory and “self-regulating” as discussed above.

### Stimulation of GABA_B_ receptor in NTS and DMV inhibition SIPSCs in SST neurons and gastric projection neurons

To assess the underlying mechanisms by which baclofen increases gastric motility on the NTS, we focused on the inhibition of identifiable SST neurons in the nucleus (Lewin et al., 2016; Thek et al., 2019). Moreover, since it is not possible to identify NTS GABA projection neurons, we examined the effect on their target gastric projection DMV neurons (Richardson et al., 2013; Lewin et al., 2016).

The sIPSCs in both SST and gastrointestinal-projection neurons of the DMV were significantly blocked by baclofen, but not completely. This suggests heterogeneity of the GABAergic inputs to these neurons, or uneven distribution of GABA_B_ receptors. The strong inhibitions of GABAergic phasic currents in gastric DMV neurons were accompanied by a consistent outward current, as previously reported (Browning and Travagli, 2001). We also observed baclofen-induced outward current in the SST neurons (Figure 4D). Our results in Figure 5 show that light-induced activation of SST neurons elicits BMR-sensitive GABAergic IPSC in target gastric DMV neurons and reciprocal IPSCs between SST neurons, as has been previously reported in the NTS (Thek et al., 2019; Bellusci et al., 2022). Baclofen inhibition of optogenetically evoked GABA-mediated IPSC was significantly larger in SST neurons than in gastrointestinal-projection DMV neurons. This observation brings further support to the uneven distribution of GABA_B_ receptors. It also may suggest that baclofen preference for the SST–SST synaptic interaction may play a substantial role as a dynamic regulator of gastric vagal circuitry.

In summary (see Figure 6), our results show that GABA_B_ signaling in SST and gastric DMV neurons increases motility *via* the release of inhibitory “brake” of NTS on excitatory gastrointestinal neuron to produce stomach contractions. Furthermore, we suggest that the increase of gastric motility is dually affected by presynaptic GABA_B_ receptors on nodose afferents and on inhibitory NTS neurons (Brooks et al., 1992) and their projection terminals on the DMV neurons, hence uncoupling the pacemaker-like gastrointestinal neurons in the DMV that project the stomach (Pedarzani et al., 2000; Richardson et al., 2013). Presumably, the differential sensitivity is essential in modulating the “on–off” activity network of the gastric vagovagal reflex in the DVC.

## Data availability statement

The raw data supporting the conclusions of this article will be made available by the authors, without undue reservation.

## Ethics statement

The animal study was reviewed and approved by Georgetown University Institutional Animal Care and Use Committee (IACUC).

## Author contributions

LB, EK, SV, and NS performed the experiments and analyzed data and prepared figures. LB, RG, SV, and NS conceived and designed the study and wrote the manuscript. All authors approved the final version of the manuscript.

## Funding

This work was funded by the National Institutes of Health (NIDDK; United States) grant R01-DK117508.

## Conflict of interest

The authors declare that the research was conducted in the absence of any commercial or financial relationships that could be construed as a potential conflict of interest.

## Publisher's note

All claims expressed in this article are solely those of the authors and do not necessarily represent those of their affiliated organizations, or those of the publisher, the editors and the reviewers. Any product that may be evaluated in this article, or claim that may be made by its manufacturer, is not guaranteed or endorsed by the publisher.
